# Mondegreens and Soramimi as a Method to Induce Misperceptions of Speech Content – Influence of Familiarity, Wittiness, and Language Competence

**DOI:** 10.1371/journal.pone.0084667

**Published:** 2014-01-08

**Authors:** Claudia Beck, Bernd Kardatzki, Thomas Ethofer

**Affiliations:** 1 Department of Biomedical Magnetic Resonance, University of Tübingen, Tübingen, Germany; 2 University Clinic for Psychiatry and Psychotherapy, University of Tübingen, Tübingen, Germany; University of Leicester, United Kingdom

## Abstract

Expectations and prior knowledge can strongly influence our perception. In vision research, such top-down modulation of perceptual processing has been extensively studied using ambiguous stimuli, such as reversible figures. Here, we propose a novel method to address this issue in the auditory modality during speech perception by means of Mondgreens and Soramimi which represent song lyrics with the potential for misperception within one or across two languages, respectively. We demonstrate that such phenomena can be induced by visual presentation of the alternative percept and occur with a sufficient probability to exploit them in neuroscientific experiments. Song familiarity did not influence the occurrence of such altered perception indicating that this tool can be employed irrespective of the participants’ knowledge of music. On the other hand, previous knowledge of the alternative percept had a strong impact on the strength of altered perception which is in line with frequent reports that these phenomena can have long-lasting effects. Finally, we demonstrate that the strength of changes in perception correlated with the extent to which they were experienced as amusing as well as the vocabulary of the participants as source of potential interpretations. These findings suggest that such perceptional phenomena might be linked to the pleasant experience of resolving ambiguity which is in line with the long-existing theory of Hermann von Helmholtz that perception and problem-solving recruit similar processes.

## Introduction

Our perception of the environment and ourselves is strongly shaped by our expectations. This is reflected in sayings such as “I did not believe my eyes” or in well-known psychological phenomena, such as the placebo effect [1], [2] and the McGurk illusion [3]. Expectations can both result in acceleration [4] as well as alteration of perception across sensory modalities. For vision, such perceptual changes have been described for judgments of emotional faces if they are preceded by affective signals expressed by speech melody [5]. Similarly, expectations can modify the perceived intensity of gustatory [6] or painful perceptions [7]. An elegant method to examine top-down modulation of perception is to employ stimuli which can induce two concurring percepts which has been extensively employed in vision research using reversible figures, such as Rubin’s vase-face illusion [8]. So far, much less is known about how expectations can alter perception in the auditory modality. For speech content, such ambiguous stimuli that can be perceived in two different ways are called ‘slips of the ear’ [9] which have been described as phenomena during which ‘a listener reports hearing, as clearly and distinctly as any correctly perceived stretch of speech, something that does not correspond to the speaker’s actual utterance’ [10]. We will focus on slips of the ear as a means to study top-down modulation of perception.

We introduce a novel approach to induce such altered perception of verbal messages by means of misheard song lyrics. This phenomenon was named “Mondegreen” in reference to its first description by Sylvia Wright for the Scottish folk song “The Bonny Earl O’Morray” [11] in which the author understood in its last line “They have slain Earl O’Morray/and Lady Mondegreen” instead of “They have slain the Earl O’Morray/and laid him on the green”. Interestingly, this phenomenon can be induced by explicitly calling the attention on passages of lyrics with the potential for misperception. In many cases, such induced “Mondegreens” are amusing for the listener and thus songs with possibly misheard passages were broadcasted by several radio stations in the recent past (for examples, please see www.youtube.com, search item: misheard lyrics).

In addition to Mondegreens which are restricted to the original language, mishearing of lyrics can also result in homophonic/near-homophonic translations into another language (typically into the native language of the listener). This phenomenon has its longest tradition in Japanese and is thus called “Soramimi” (which means mishearing in Japanese). However, Soramimi became recently also popular in other cultures and have been named after prototypical homophonic translations within their respective language: In German, Soramimi are called “Agathe Bauer” a combination of a German first and family name with “Bauer” meaning “farmer” misheard from the song of Snap “I got the power”. In Dutch, this phenomenon became popular under the name “Mama Appelsap” (meaning momma apple juice) misheard from a passage of Michael Jackson’s song “Wanna be starting something”.

In spite of the popularity of Mondegreens and Soramimi with regular radio and television broadcasting devoted to this topic, no scientific studies have been conducted to examine this phenomenon. This is even more surprising as people often report that the altered perception can be quite persistent occurring every time the song is heard, making Mondegreens and Soramimi a valuable tool to induce plasticity within the auditory system. It is important to acknowledge, however, that the potential of induction of both Mondegreen and Soramimi is variable across the employed songs as well as listeners. Therefore, the first step towards establishing these phenomena as method for induced misperceptions is to investigate the variability depending on stimulus-bound as well as interindividual influence factors for the occurrence of within-language (Mondegreen) and across-language (Soramimi) misperceptions. In the current study, we focused on the familiarity of the participants with the employed song texts as well as prior knowledge and rated wittiness of the misperceived lyrics. We had no a priori hypothesis whether familiarity with the original song texts make the participants more persistent against or susceptible for induced misperceptions and thus tested possible influences of this factor using a bidirectional hypothesis. As it is often reported that such altered perception is stable across time, we expected stronger effects if the alternative lyrics were already known to the study participants. Emotion has been repeatedly demonstrated to have a strong impact on encoding of novel information (e.g., [12], [13]). Therefore, we hypothesized that the joy typically induced by Mondegreens/Soramimi would result in enhanced encoding of alternative lyrics and thus a positive relationship between the strength on induced misperceptions and their wittiness was expected. Finally, we investigated the impact of verbal fluency and linguistic competence on occurrence of such phenomena. We predicted that high verbal fluency within the native language of the participants is associated with increased occurrence of within-language misperceptions as listeners with high verbal fluency might use their broad vocabulary to compensate ambiguous perception caused by inaccuracies in the pronunciation of the singer. In contrast, competence for the language of the songs with potential Soramimi (English) was expected to be a protective factor against across-language misperceptions into the native language of the participants (German) as we hypothesized that such misperceptions are partly driven by the fact that unknown foreign words are automatically replaced by known words of the native language of the listener.

## Materials and Methods

### Ethics Statement

The study was approved by the Ethical Committee of the University of Tuebingen (votum: 215/2012 BO2) and written informed consent was obtained from all participants. All study procedures were in line with the latest version of the Declaration of Helsinki.

### Participants

23 healthy German native speakers (12 women, 11 men, mean age: 28.3±6.8 years, education: 16.4±2.8 years) participated in this study. All participants were right-handed according to the Edinburgh Handedness Inventory [14]. Mean verbal intelligence of the participants as obtained by a German vocabulary test (Mehrfach-Wortschatz-Intelligenztest B) was 112.9±11.5. Knowledge of English language was assessed using a language test based on a short form of the Test of English as a Foreign Language (Mini-TOEFL, http://www.ets.org/toefl) to determine the participants’ grammar and vocabulary comprehension. On average, the participants correctly answered 15.4±5.1 out of 25 questions. Verbal intelligence and English language comprehension were significantly correlated (r = .67, two-tailed p<.01) reflecting the fact that they are similarly driven by the educational level of the participants. All values are given in mean ± standard deviation.

### Stimuli

The stimulus set comprised short audio clips (mean duration: 17.7±3.0 sec) taken from 41 English and 20 German songs which were broadcasted by radio stations because of their potential to induce within- or across language misperceptions. All stimuli were normalized to same peak intensity.

### Experimental Design

All stimuli were presented during three consecutive experimental runs in a fully randomized order. During experimental run 1, the participants judged their degree of familiarity with the songs on a four-point scale (‘unknown’, ‘know melody’, ‘know refrain’, ‘know text’) that was visually presented for five seconds after stimulus offset. After this judgment period, a fixation cross was shown for two seconds. Before starting experimental run 2, the participants were informed that the song texts can be misheard and that such alternative lyrics will be presented visually during the next experimental run. The task of the participants was to determine whether these alternative lyrics were already known to them (because they heard them in radio transmissions or even spontaneously misheard the lyrics in the presented manner) during a four second period which was followed by a presentation of a fixation cross for two seconds. In experimental run 3, participants were instructed to judge whether and how strongly they misheard the lyrics as presented visually during the second run on a four point scale (‘not at all’, ‘slightly’, ‘moderate’, ‘strongly’). It was emphasized during instruction of the volunteers that the task is not to indicate whether they remember the alternative lyrics, but to indicate to what extent an alteration of perception occurs. Directly after judging the strength of possible misperceptions, the participants rated the wittiness of these misperceptions on a four point scale (‘not at all’, ‘slightly’, ‘moderate’, ‘strongly’). For both ratings in this third experimental run, participants conveyed their decision during a four second interval and a fixation was shown for two seconds before the next stimulus was presented.

### Data Analysis and Hypotheses

All values are given in mean ± standard error of the mean (SEM) unless otherwise specified. For correlation coefficients, we employed back-transformed mean and SEM values of individual Fisher Z scores.

We first determined the frequency and the variability (i.e., range and standard deviation, sd) of misperceptions across the employed stimuli as well as the study participants separately for within- and between-language misperceptions. We then tested whether familiarity with the employed songs as well as prior knowledge and wittiness of the alternative lyrics influenced the strength of induced misperceptions. In addition, we determined potential influences of interindividual differences in verbal intelligence and knowledge of English on these misperceptions.

#### Effect of familiarity (experimental run 1)

We calculated individual correlation coefficients between song familiarity and strength of misperceptions separately for each subject. These individual correlation coefficients were then transformed to Fisher Z scores. As we had no a priori assumption on the direction of the effect of familiarity (bidirectional hypothesis), we subsequently submitted these values to a two-tailed one-sample t-test.

#### Effect of prior knowledge of alternative lyrics (experimental run 2)

We hypothesized that prior knowledge (i.e., previous encounters with the alternative lyrics in the media) increases the frequency of misperceptions (unidirectional hypothesis). To test this hypothesis, we statistically compared the frequency for misperceptions of previously known versus unknown alternative song texts using one-tailed paired t-tests. In this analysis, only participants (17 for within- and 20 for across-language trials) who knew at least one alternative song text of the respective types of misperceptions could be included (otherwise the frequency in previously known misperceptions cannot be defined due to a division by zero in these subjects).

In addition, we determined whether subjects who already know many alternative song texts are generally prone to such misperceptions. To this end, we investigated whether the probability to misperceive songs in which the alternative lyrics were not known to the subject increases with the amount of previously known alternative song texts by means of a correlation analysis (unidirectional hypothesis).

#### Effect of wittiness of alternative lyrics (experimental run 3)

We calculated individual correlation coefficients between rated wittiness and strength of misperceptions separately for each subject. As the subjects judged the wittiness of perceived misperceptions, only those trials during which the subjects reported to have misperceived the lyrics were included in this analysis (8.5±0.8 within-language trials, 13.7±0.1 across-language trials). Furthermore, as correlation analyses require data that are distributed at least across two factor levels for both investigated variables, data of one participant had to be excluded for the across-language misperceptions and data of five participants had to be excluded from the within-language misperceptions for this analysis.

The individual correlation coefficients were then transformed to Fisher Z scores. As we hypothesized a positive relationship between these factors (unidirectional hypothesis), we subsequently submitted these values to a one-tailed one-sample t-test.

#### Effect of verbal intelligence and knowledge of English

We additionally evaluated whether verbal intelligence as well as knowledge of English influenced misperceptions. To this end, mean values of ratings for misperceptions were calculated separately for within- and across-language trials for each subject and correlation analyses of these values with verbal intelligence scores as obtained by the MWTB as well as correct responses in the Mini-TOEFL-test were conducted. As we predicted that high verbal fluency increases the occurrence of within-language misperceptions while linguistic competence in English protects against across-language misperceptions, we employed one-tailed t-tests to address these directional hypotheses.

## Results

All data are freely available and can be downloaded as supplemental material (Data S1). Frequencies of normal song perception and misperceptions (slight, moderate, and strong) as well as missed responses are presented in Table 1 separately for within- and across-language trials. Subjects responded in more than 99% of the trials indicating that the four second period was sufficient to judge the strength of misperceptions. Within-language misperceptions occurred with a significantly higher frequency than across-language misperceptions (42.6% ±3.8% versus 33.5% ±3.8%, t(22) = 2.62, two-tailed p<.05). For both types of misperceptions, a large variability was found across the employed stimulus material ranging from 8.7% to 87.0% (sd: 20.3%) for within- and 4.4% –82.6% (sd: 21.1%) for between-language misperceptions. Similarly, these frequencies also varied considerably across participants ranging from 15.0% to 90% (sd: 18.3%) for within- and 9.8% to 85.4% (sd: 18.1%) for across-language misperceptions.

**Table 1:** Percentage of within and across language misperceptions.

Strength of misperception	Within-language	Across-language
None	56.7%±3.8%	66.0%±3.8%
Slight	17.0%±2.7%	11.0%±1.5%
Moderate	10.7%±2.0%	11.5%±1.7%
Strong	15.0%±2.8%	11.0%±2.2%
Missed response	0.7%±0.4%	0.5%±0.3%

All values are given in mean ± standard error.

We then tested whether the familiarity of the participants with the stimulus material (experimental run 1), prior knowledge of the alternative lyrics (experimental run 2) and wittiness of these altered song texts (experimental run 3) influenced the strength of misperceptions.

For familiarity with the song texts as obtained in experimental run 1, no significant correlation was found for either within- (r = .08±.04, t(22) = 2.00, two-tailed p = .06) or across-language misperceptions (r = −.01±.03, t(22) = −0.20, two-tailed p = .85).

Prior knowledge of the alternative song texts as assessed in experimental run 2, however, was strongly predictive for both within- (72.1% ±9.6% versus 40.5% ±3.7%, t(16) = 2.78, one-tailed p<.05 for known versus unknown alternative lyrics, respectively) and across-language misperceptions (74.0% ±6.2% versus 30.0±4.0%, t(19) = 7.08, one-tailed p<.001 for known versus unknown alternative lyrics, respectively) in experimental run 3. However, prior knowledge cannot explain the generally higher frequency of within- than across-language misperceptions as the participants were familiar with more alternative song texts for across- than within-language trials (13.5% ±2.8% versus 6.7±1.2%, t(22) = 2.40, two-tailed p<.05). Furthermore, previous knowledge of alternative lyrics did not generally increase the probability of misperceptions as no significant correlation was found between the number of known alternative song texts and the probability to misperceive songs for which the alternative lyrics were not known to the subject (r = .03, one-tailed p = .44 for within- and r = .07, one-tailed p = .38 for across-language misperceptions).

We also asked whether the strength of misperceptions was influenced by the extent the participants perceived them as witty. This was indeed the case as the mean within-subject correlation coefficient for ratings of the strength of misperceptions and judgments of their wittiness was r = .55±.13 (t(17) = 4.23, one-tailed p<.001) for within- and r = .58±.09 (T(21) = 6.31, one-tailed p<.001) for across-language misperceptions.

Finally, we investigated possible influences of verbal intelligence and comprehension of English language on misperceptions. In agreement with our a priori hypothesis, the mean rating of misperceptions in within-language trials calculated separately for each subject correlated significantly with verbal intelligence (r = .46, one-tailed p<.05) of the participants. For across-language trials, however, the effect was opposite to our hypothesis as language competence as assessed with the Mini-TOEFL was also positively correlated with mean ratings for across-language trials, but failed to reach significance (r = .28, one-tailed p = .10).

## Discussion

Induced alterations of perception as an experimental tool have a long-standing tradition in research within the visual domain (e.g. reversible figures, such as the Rubin’s vase-face illusion [8]) and have been successfully employed to study cerebral processing of perceptual phenomena [15], [16]. The primary strength of paradigms relying on such induced changes in perception is that they elegantly enable investigation of top-down influences (e.g., prior expectations, memory) without changing bottom-up parameters (i.e., physical parameters of the stimulus material). A principal advantage of examining altered perception in the auditory modality is that acoustic information is expressed in the temporal domain which offers the opportunity to exactly pinpoint the onset of events with altered perception. Thus, studying such phenomena in the auditory domain enables to overcome methodological obstacles typically encountered during visual presentation of reversible figures that induce spontaneous as well as unpredictable switches between two competing percepts without sufficient temporal stability necessitating modification of the stimuli, such addition of embossing [15].

In the current study, we propose a novel method for targeted induction of auditory misperceptions by means of Mondegreens/Soramimi. To this end, we visually presented the alternative lyrics during listening to the respective song parts and asked the participants to rate the strength of misperceptions in a succeeding run without additional visual cue. Altered perception occurred in 42.6% of the within- and 33.5% of the across-language trials. These results indicate that careful stimulus selection based on the current evaluation experiments would allow obtaining a set of stimuli for which altered perception can be expected in about half of the trials. A probability of 50% would be optimal for application of these stimuli in a classical 2×2 factorial design with induction (before versus after induced misperceptions) and perception (original versus alternative lyrics) as within-subject factors during neuroimaging studies.

We also evaluated potential stimulus-dependent and interindividual influence factors that might predict the occurrence of such phenomena. Familiarity with the stimulus material has been shown to have a profound impact on neural processing of musical stimuli [17]. In our study, the correlation between the familiarity of listeners with the presented musical stimuli and the occurrence of altered perception was close to zero indicating that these phenomena can be studied without potential biases of song familiarity. On the other hand, previous knowledge of the alternative lyrics strongly influenced whether or not misperceptions occurred which is in line with the reports of many of our study participants that induced misperceptions can result in long-lasting effects that occur each time the respective song is heard. Thus, it might be advisable to obtain data on familiarity with the alternative lyrics to be able to exclude such trials in neuroscientific experiments relying on a design comparing responses before versus after induced misperceptions.

A large body of evidence indicates greater allocation of attentional resources [18], enhancement of perceptual vividness [19], as well as better memory [20] to stimuli of affective value and similar effects have also been demonstrated for musical stimuli [21]–[23]. Therefore, we predicted that humor appreciation modulates the occurrence of induced misperceptions. Indeed, the rated wittiness of the alternative lyrics correlated significantly with the intensity of misperceptions explaining about 30% of the variance. This finding is in line with observations made in the visual domain where resolving ambiguities of visual percepts has been found to generate pleasant feelings similar to the experience of problem solving [24]. These findings underline the importance of affective modulation in perceptual processes including the induced misperceptions examined here and suggest that future studies of such phenomena should encompass ratings of wittiness to study the neural correlates underlying modulation of such perceptual phenomena.

In concordance with our a priori hypothesis, a positive relationship between verbal fluency in German on the one hand and the strength of induced within-language misperceptions on the other hand was found. This finding indicates that the more exhaustive vocabulary of participants with high verbal fluency can be automatically used to generate alternative solutions in ambiguous perceptual situations. Regarding across-language misperceptions, however, we did not find the expected negative, but rather a positive relationship for linguistic competence as assessed by the Mini-TOEFL. This finding has to be interpreted with caution as it failed to reach significance, but at least argues clearly against the assumption that such mishearings are simply driven by inadequate language knowledge of the listeners [25].

In summary, we propose a novel method for investigating top-down effects on processing of language stimuli by means of induced Mondegreens/Soramimi as experimental tool. We demonstrated that such phenomena occur with a sufficient frequency for application in neuroscientific experiments that aim to study processing of ambiguous stimuli and how such processing is modulated by expectations. Interestingly, the occurrence of induced misperceptions was independent of knowledge of the original, but not of the alternative percept which is in line with observations made in the visual domain for reversible figures demonstrating that we can get stuck in one interpretation until we are informed that there is an alternative interpretation. Finally, wittiness of the misherard lyrics as stimulus-dependent and verbal fluency as interindividual factor increased the strength of induced misperceptions indicating that this phenomenon depends on whether the alternative percept was experienced as pleasant as well as on the vocabulary of the participants as source of possible interpretations. These findings suggest that these phenomena are linked to the joyful feeling of resolving ambiguity and concur with the long-existing theories formulated by Hermann von Helmholtz who hypothesized that perception relies on similar processes as intellectual problem solving [26].

## Supporting Information

Data S1 **The supplemental data file includes information on analog data of the study participants as well as data analyses according to the subjects and stimuli (familiarity with the song text, familiarity with misperceptions, strength of misperceptions, wittiness uncorrected and corrected).**
(XLSX)
